# A novel Anti-ROS osteoblast-specific delivery system for ankylosing spondylitis treatment via suppression of both inflammation and pathological new bone formation

**DOI:** 10.1186/s12951-023-01906-2

**Published:** 2023-05-26

**Authors:** Guan Zheng, Xiaoshuai Peng, Yunhui Zhang, Peng Wang, Zhongyu Xie, Jinteng Li, Wenjie Liu, Guiwen Ye, Yucong Lin, Guojian Li, Huatao Liu, Chenying Zeng, Lihua Li, Yanfeng Wu, Huiyong Shen

**Affiliations:** 1 Department of Orthopedics, The Eighth Affiliated Hospital, Sun Yat-sen University, 3025# Shennan Road, Shenzhen, P.R. China; 2 Center for Biotherapy, The Eighth Affiliated Hospital, Sun Yat-sen University, 3025# Shennan Road, Shenzhen, P.R. China; 3 Department of Applied Physics, The Hong Kong Polytechnic University, Hung Hom, Kowloon, Hong Kong P.R. China; 4 Future Technology Research Institute, South China Normal University, 55 Zhongshan Dadao, Tianhe District, Guangzhou, P.R. China

**Keywords:** MnFe2O4 nanoparticles, Ankylosing spondylitis, ROS scavenging, Targeted therapy, Nanomedicine

## Abstract

Ankylosing spondylitis (AS) is a common rheumatic disorder distinguished by chronic inflammation and heterotopic ossification at local entheses sites. Currently available medications, including nonsteroidal anti-inflammatory drugs (NSAIDs), disease-modifying anti-rheumatic drugs (DMARDs) and TNF inhibitors, are limited by side effects, high costs and unclear inhibitory effects on heterotopic ossification. Herein, we developed manganese ferrite nanoparticles modified by the aptamer CH6 (CH6-MF NPs) that can efficiently scavenge ROS and actively deliver siRNA into hMSCs and osteoblasts in vivo for effective AS treatment. CH6-MF NPs loaded with BMP2 siRNA (CH6-MF-Si NPs) effectively suppressed abnormal osteogenic differentiation under inflammatory conditions in vitro. During their circulation and passive accumulation in inflamed joints in the *Zap70*^*mut*^ mouse model, CH6-MF-Si NPs attenuated local inflammation and rescued heterotopic ossification in the entheses. Thus, CH6-MF NPs may be an effective inflammation reliever and osteoblast-specific delivery system, and CH6-MF-Si NPs have potential for the dual treatment of chronic inflammation and heterotopic ossification in AS.

**Supplementary Information:**

The online version contains supplementary material available at 10.1186/s12951-023-01906-2.

## Introduction

Ankylosing spondylitis (AS), which affects 0.1–0.5% of the population worldwide, [1] is an intractable rheumatic disorder distinguished by chronic inflammation and pathological ossification at local sites of entheses [2]. Currently, the main goal of clinical treatment strategies is to control inflammation and halt the progressive development of disease using medications, including nonsteroidal anti-inflammatory drugs (NSAIDs), disease-modifying antirheumatic drugs (DMARDs), [3] and TNF inhibitors [4–7]. In addition to the unwanted side effects and high costs associated with these treatments, [8–10] whether these drugs can inhibit pathological new bone formation remains to be proven [11–15]. Therefore, new treatment strategies are still urgently needed.

Currently, the main difficulty in the treatment of AS is its unclear pathogenesis, especially the unclear relationship between inflammation and pathological ossification. Human mesenchymal stem cells (hMSCs) have essential functions in both bone metabolism and immune homeostasis due to their strong immunoregulatory and osteogenic differentiation abilities [16–19]. Previously, we found that the improved osteogenic differentiation capability of hMSCs, confirmed by higher expression levels of BMP-2 both in vitro and in vivo, may be a potential mechanism of abnormal new bone formation in AS [20, 21].

TNF-α, a major proinflammatory cytokine, has a crucial function in the pathogenesis of AS. Our studies, [22] as well as the results of Li et al. [23] and Yang and Dai, [24] indicated that TNF-α promotes osteogenic differentiation, directional migration of hMSCs, and abnormal new bone formation in AS. Furthermore, TNF-α triggers intracellular ROS generation, [25, 26] and the interaction between ROS and TNF-α promotes the development of inflammation and disease [26].

Catalytic nanoparticles, also known as nanozymes, are nanomaterials with enzyme-catalysed capabilities. Because of their low cost, easy preparation, controllable size and adjustable function, the therapeutic potential of nanozymes, such as Au-Bi_2_Se_3_ NPs, [27] Ag NPs, [28] MnO_2_ NPs, [29] manganese ferrite [30] and ceria NPs, [31] as effective scavengers of ROS for the treatment of various inflammatory diseases has recently received increasing attention.

An abnormal vascular system and inflammatory cell infiltration lead to a significant increase in local vascular permeability at inflamed sites, which enables nanoparticles to passively accumulate at these sites, and this effect is called the extravasation through leaky vasculature and subsequent inflammatory cell-mediated sequestration (ELVIS) effect [32]. Moreover, through modification by nucleic acid aptamers, nanoparticles can actively target specific tissues or cells. Nucleic acid aptamers are screened nucleic acids with unique 3D structures that can specifically bind to target molecules. This feature enables the application of aptamers for various biomedical purposes, including cell-targeted therapy [33].

However, as mentioned above, a single treatment strategy, such as TNF inhibitors, may not be effective for the treatment of AS due to its complex pathological mechanism. RNA interference (RNAi) has been considered an efficient tool to silence the expression of targeted genes for the treatment of genetic diseases, including cancer, [34] autoimmune disorders, [35] and metabolic diseases [36]. In contrast to traditional therapies, siRNA-based therapy can maintain high therapeutic specificity while avoiding side effects, and it can be implemented via nanomaterial drug delivery systems, which can enhance the specificity of the siRNA for the target tissues and cells.

**Scheme 1:**
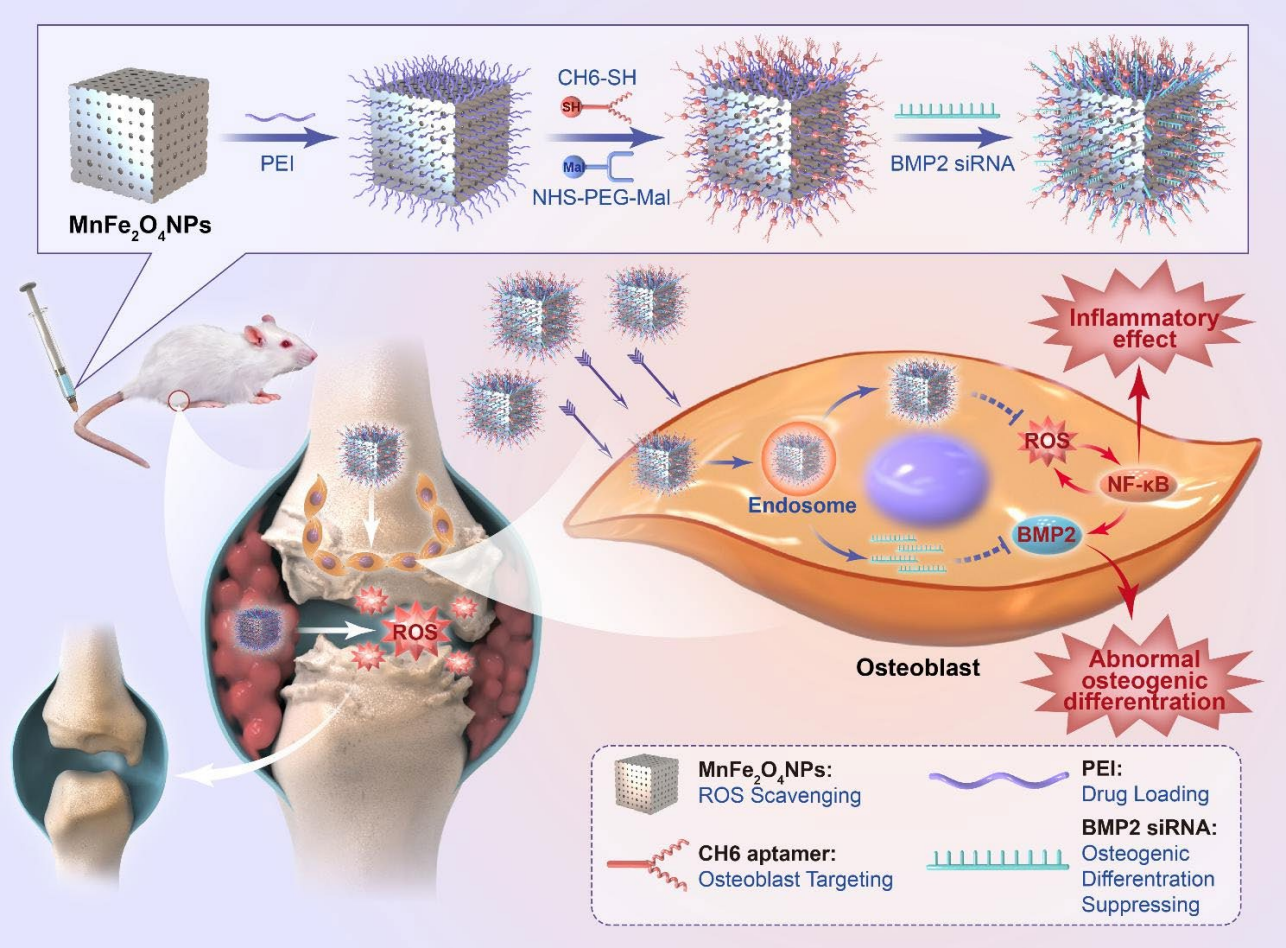
Schematic illustration of the preparation of CH6-MF-Si NPs and their application in AS therapy

In the current work, we designed an anti-ROS osteoblast-specific delivery system based on manganese ferrite nanoparticles. After modification with polyethylenimine (PEI) and the osteoblast-specific aptamer CH6, [37] CH6-MF NPs could target specific inflammatory sites in AS, scavenge ROS and alleviate inflammation. Moreover, CH6-MF NPs could be loaded with BMP2 siRNA (CH6-MF-Si NPs) and achieve osteoblast-specific delivery of siRNA, thus suppressing abnormal new bone formation in AS (Scheme 1). The cell-specific delivery ability and therapeutic potential of the nanoparticles were examined in vitro and in vivo. We further studied the effects of CH6-MF NPs on abnormal osteogenic differentiation under inflammatory conditions. In this work, we constructed a unique anti-ROS osteoblast-specific delivery nanomaterial for the dual treatment of chronic inflammation and heterotopic ossification in AS.

## Results and discussion

### Synthesis and characterization of CH6-MF NPs

**Fig. 1:**
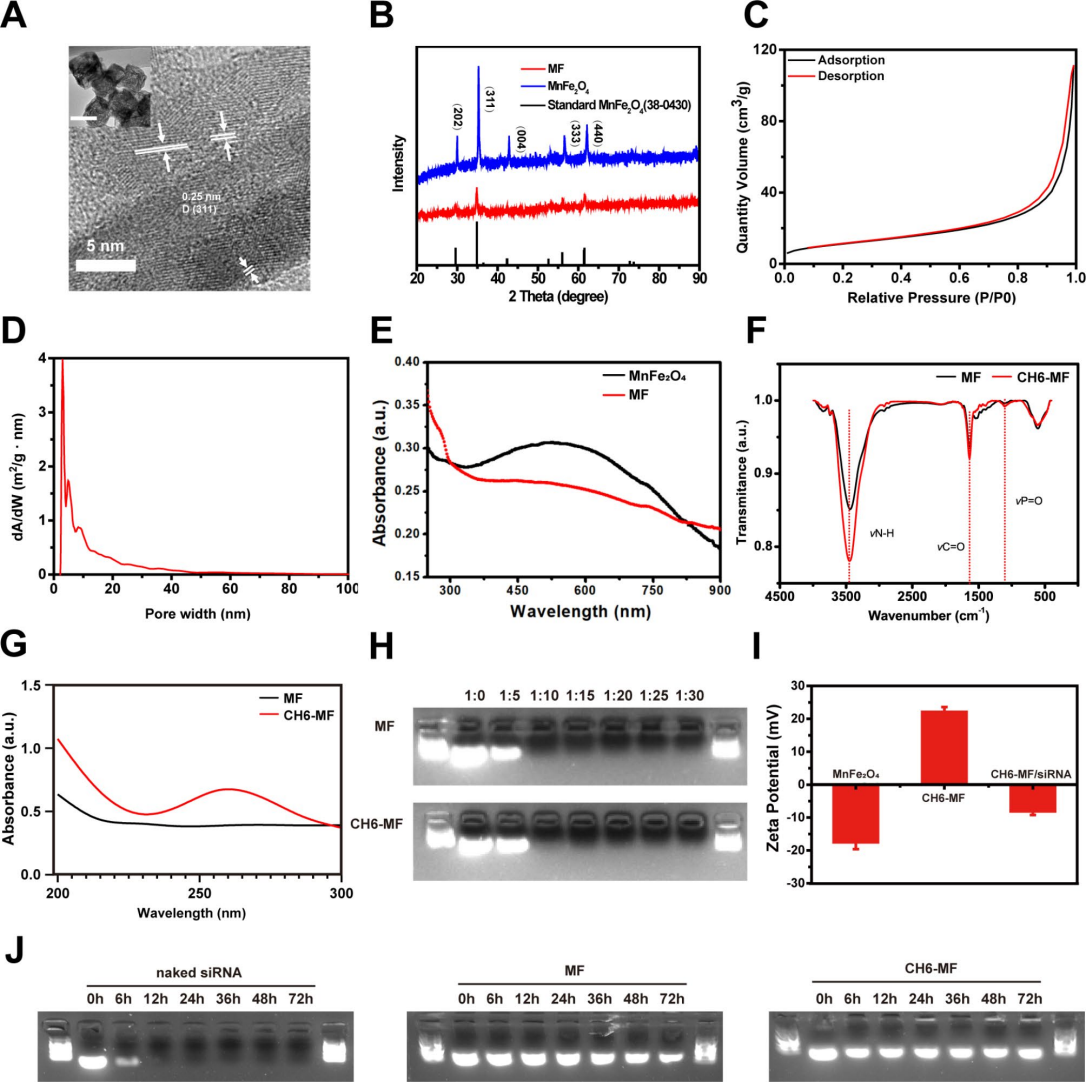
Characterization of MF NPs and CH6-MF NPs. (A) HRTEM image of synthesized MnFe_2_O_4_ NPs. The NPs exhibited a large number of surface defects with a twisted lattice, dislocations and distortions (inset: TEM image of synthesized MnFe_2_O_4_ nanoparticles, scale bar = 100 nm). (B) XRD spectrum of synthesized MnFe_2_O_4_ nanoparticles, MF NPs, and tetragonal standard MnFe_2_O_4_. (C) N_2_ adsorption/desorption isotherms of synthesized MnFe_2_O_4_ NPs. (D) Pore size distribution of synthesized MnFe_2_O_4_ NPs with abundant surface defects. (E) UV–vis spectra of synthesized MnFe_2_O_4_ NPs and MF NPs. (F) FTIR spectra of MF NPs and CH6-MF NPs. (G) UV–vis spectra of MF NPs and CH6-MF NPs from 200 to 300 nm. (H) Agarose gel electrophoresis (110 V, 5 min) of NP-siRNA complexes at different weight ratios. (I) Zeta potential variations of synthesized MnFe_2_O_4_ NPs, CH6-MF NPs and CH6-MF NPs/siRNA. (J) siRNA serum stability of naked siRNA, MF NPs/siRNA and CH6-MF NPs/siRNA complexes(NP:siRNA weight ratio of 10:1) incubated with fetal bovine serum(50% final concentration) at 37 °C for different times. Data were analysed by Student’s t test and one-way ANOVA. The outcomes are presented as the mean ± Sd. ns = statistically nonsignificant, *P < 0.05, **P < 0.01, and ***P < 0.001; n = 3

MnFe_2_O_4_ NPs with abundant surface defects were fabricated by a facile one-step thermal chemical approach. The TEM image in the inset of Fig. 1A illustrates the cubic and highly porous structure of the synthesized MnFe_2_O_4_ NPs. The twisted lattice, many dislocations and distortions in Fig. 1A demonstrate the highly defect-rich edges. The main lattice spacing of 0.256 nm in the HRTEM image (Fig. 1A) was consistent with the main (311) peak in the XRD spectrum (Fig. 1B). Moreover, the XRD spectra of MnFe_2_O_4_ NPs and MF NPs corresponded well with the tetragonal standard MnFe_2_O_4_ (PDF 38–0430). To further prove the porous structure shown in Fig. 1A, nitrogen adsorption-desorption isotherms (Fig. 1C and D) were employed to study the structure of the synthesized MnFe_2_O_4_ nanoparticles. The results showed the highly mesoporous structure of the NPs, with a large surface area (41.2205 m²/g) and an appropriate pore volume (0.172 cm^3^ g^− 1^). The average pore diameter was calculated to be 17.048 nm. After modification with PEI, the absorption spectra (Fig. 1E) illustrated the successful modification of the MnFe_2_O_4_ surface with amino groups. As shown in Fig. 1F, Fourier transform infrared spectroscopy (FTIR) was employed to examine the chemical bonds in CH6-MF NPs. Compared with those for MF NPs, the stronger absorption peaks at 3452, 1640, and 1105 cm^-1^ for CH6-MF NPs can be attributed to the stretching vibrations of N-H, C = O, and P = O, respectively, which illustrated the successful modification of the MF NPs with the aptamer CH6. The strong absorption peak at 260 nm in the absorption spectra for CH6-MF NPs (Fig. 1G) also demonstrated successful modification with the osteoblast-specific aptamer CH6. Both the MF NPs and CH6-MF NPs possessed positive charges after PEI modification, which implied that both nanoparticles had the ability to deliver negatively charged siRNA. The siRNA binding ability of MF NPs and CH6-MF NPs was investigated using agarose gel electrophoresis. In our experiments, we investigated weight ratios ranging from 5 to 30. When the weight ratio of MF NPs/siRNA reached 10:1, the migration of siRNA in the agarose gel was completely blocked, indicating that siRNA could be completely bound by the MF NPs. Meanwhile, CH6 aptamer modification of CH6-MF NPs had negligible effects on their binding ability (Fig. 1H). Thus, the NP-siRNA complexes at a NP:siRNA weight ratio of 10:1 were selected for subsequent experiments. The surface zeta potential changes of the nanoparticles also confirmed their strong binding ability (Fig. 1I). After modification, the surface zeta potential shifted from − 17.5 mV to + 22.1 mV, ensuring that CH6-MF NPs could bind and transfer the target siRNA for further application. After mixing the NPs with siRNA for 20 min, the zeta potential changed to ~-8 mV, demonstrating that the siRNA was successfully bound to the CH6-MF NPs. To investigate whether PEI-capped nanoparticles could protect siRNA from degradation during systemic circulation, MF NPs/siRNA and CH6-MF NPs/siRNA were incubated with 50% FBS at 37 °C. As shown in Fig. 1J, naked siRNA was degraded completely within 12 h. However, both MF NPs/siRNA and CH6-MF NPs/siRNA complexes were more stable than naked siRNA, in which siRNA could survive beyond 72 h in the presence of serum. These results indicated that both MF NPs and CH6-MF NPs could protect siRNA from nuclease degradation during circulation.

### ROS scavenging of MF NPs and CH6-MF NPs

**Fig. 2:**
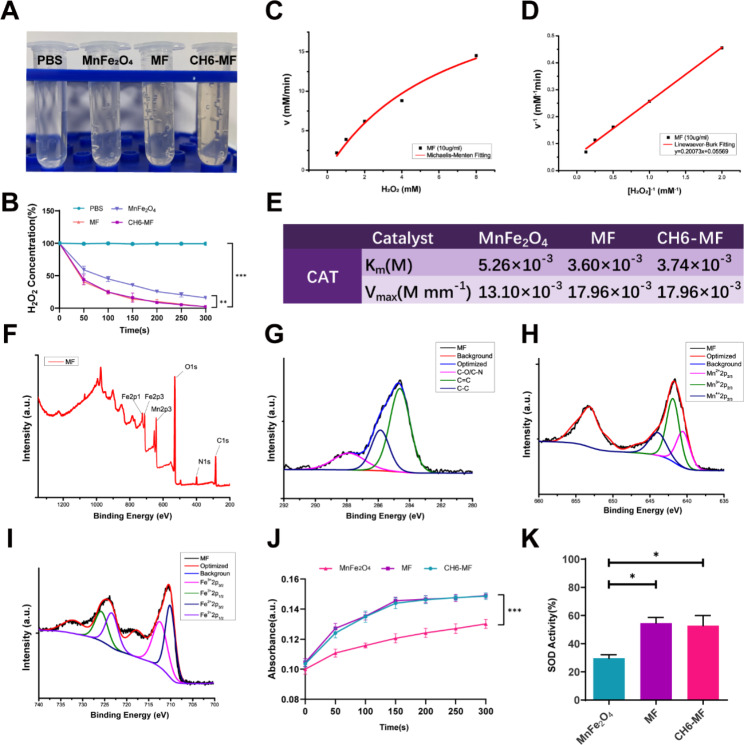
ROS scavenging ability of MF NPs. (A) Images of O_2_ generation after 5 min of incubation with 2 mM H_2_O_2_ in different groups. (B) A time-dependent H_2_O_2_ decomposition assay performed over 5 min. (C) Michaelis − Menten kinetic analysis and (D) Lineweaver − Burk plot of the CAT-like activity of MF NPs with H_2_O_2_ as the substrate at 25 °C. (E) Michaelis − Menten constant (Km) and maximum reaction rate (Vmax) of commercial MnFe_2_O_4_, MF NPs, and CH6-MF NPs with H_2_O_2_ as the substrate. (F) XPS full-scan spectrum of MF NPs. (G) XPS high-resolution scans of C 1s of MF NPs. (H) XPS high-resolution scans of Mn 2p of MF NPs. (I) XPS high-resolution scans of Fe 2p of MF NPs. (J) Peroxidase (POD) activities of commercial MnFe_2_O_4_, MF NPs, and CH6-MF NPs at a concentration of 10 µg/ml. (K) Superoxide dismutase (SOD) activities of commercial MnFe_2_O_4_, MF NPs, and CH6-MF NPs at a concentration of 10 µg/ml. Student’s t test and one-way ANOVA were used to analyse the data. The outcomes are presented as the mean ± SD. ns = statistically nonsignificant, *P < 0.05, **P < 0.01, and ***P < 0.001; n = 3

ROS are major inducers and components of inflammatory disorders, and they also promote AS pathogenesis. Here, we investigated the ROS scavenging ability of the NPs by evaluating their oxidoreductase-like activity. First, we measured the CAT-like activities of the NPs by evaluating H_2_O_2_ decomposition. In a time-dependent H_2_O_2_ assay, the control group with a mixture of H_2_O_2_ and PBS had a constant absorbance at 405 nm for 5 min. After incubation for 5 min, MF NPs and CH6-MF NPs generated more O_2_ bubbles than the commercial MnFe_2_O_4_ powders (Fig. 2A). Notably, H_2_O_2_ was completely decomposed by MF NPs and CH6-MF NPs (Fig. 2B), while nearly 15.97% of H_2_O_2_ remained in the commercial MnFe_2_O_4_ group, indicating that both MF NPs and CH6-MF NPs exhibited stronger O_2_ generation and CAT activity than MnFe_2_O_4_ nanoparticles. This high catalytic efficiency may be attributed to the defect-rich structure of MF NPs and CH6-MF NPs [38]. We further investigated the enzymatic reaction rates of NPs (10 µg/mL) by using different concentrations of H_2_O_2_ (8, 4, 2, 1, and 0.5 mM). The reaction rates were fitted with the Michaelis − Menten equation and the Lineweaver − Burk double reciprocal curve. The Michaelis − Menten curve and Lineweaver − Burk double reciprocal curve show that the Km and Vmax values of the commercial MnFe_2_O_4_, MF NPs and CH6-MF NPs with H_2_O_2_ were 5.26 × 10^− 3^ M and 13.10 × 10^− 3^ M min^− 1^ (Figure S1A and B), 3.60 × 10^− 3^ M and 17.96 × 10^− 3^ M min^− 1^ (Fig. 2C and D), and 3.74 × 10^− 3^ M and 17.96 × 10^− 3^ M min^− 1^ (Figure S1C and D), respectively, indicating a higher affinity of MF NPs and CH6-MF NPs for H_2_O_2_ (Fig. 2E). Next, X-ray photoelectron spectroscopy (XPS) technology was employed to determine the elemental composition and valence states of Mn and Fe in MF NPs. As shown in Fig. 2F, there were five typical peaks of C 1s, O 1s, N 1s, Mn 2p, and Fe 2p, confirming the elemental composition of MF NPs. Figure 2G shows that the C 1s spectrum could be deconvoluted into three components; 284.62, 285.86 and 287.86 eV were attributed to C = C, C-C, and C-O/C-N, respectively. In the Mn 2p spectrum (Fig. 2H), the peaks at 640.54, 641.89 and 643.96 eV were assigned to the Mn 2p_2/3_ features of Mn^2+^, Mn^3+^ and Mn^4+^. As shown in Fig. 2I, the peaks at 709.97 and 712.68, 722.89 and 725.53 eV are assigned to the Fe 2p_3/2_ and Fe 2p_1/2_ features of Fe^2+^ and Fe^3+^, respectively. The multivalence states of Mn and Fe confirmed the structural basis for the redox reaction.

Next, we studied the POD-like activities of MF NPs and CH6-MF NPs. Notably, after adding MF NPs or CH6-MF NPs at a final concentration of 10 µg/mL to the H_2_O_2_ and TMB mixture, the absorbance increased obviously during the reaction, and the curves were steeper than that of MnFe_2_O_4_, suggesting that both MF NPs and CH6-MF NPs possessed more significant POD-like activities than commercial MnFe_2_O_4_ nanoparticles (Fig. 2J).

In addition to CAT and POD activities, we further measured SOD activities. The SOD-like abilities of the NPs were evaluated with a WST-1 assay kit (Nanjing Jiancheng Bioengineering Institute, China). The SOD activities of MF NPs and CH6-MF NPs were 54.64% and 52.80%, respectively, which was much higher than that of commercial MnFe_2_O_4_ (29.67%) (Fig. 2K).

### Cellular Uptake, cytotoxicity and intracellular distribution of CH6-MF NPs

**Fig. 3:**
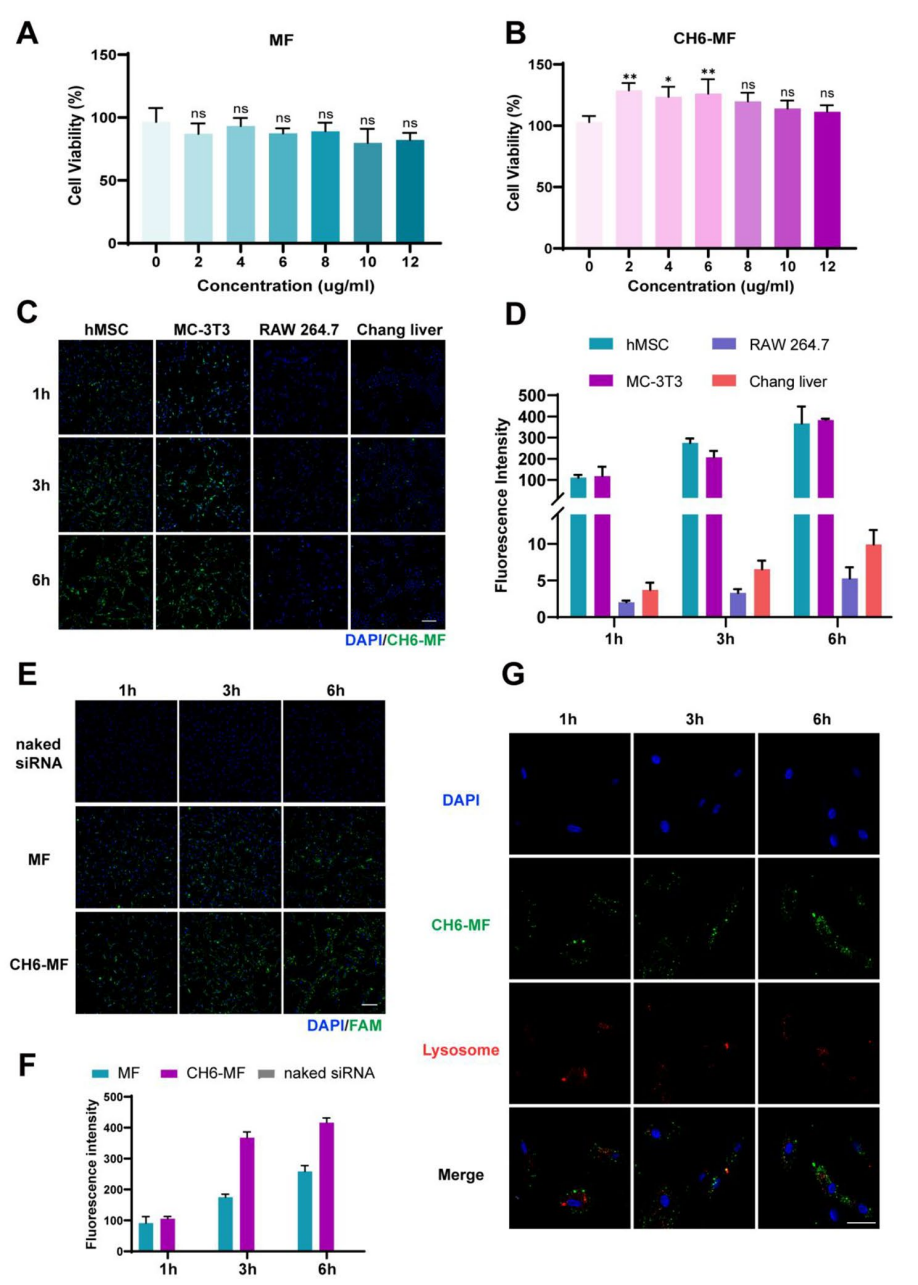
Cell viability and distribution of the nanoparticles in vitro. Viability of hMSCs after treatment with (A) MF NPs and (B) CH6-MF NPs at different concentrations for 3 days. (C) Cellular uptake of CH6-MF NPs/FAM-labelled siRNA (green) in hMSCs, MC-3T3 cells, RAW264.7 cells and Chang liver cells at different time points. The nuclei were stained with DAPI (blue). (D) Quantification of the fluorescence intensity in (C). (E) Cellular uptake of naked FAM-labelled siRNA, MF NPs/FAM-labelled siRNA and CH6-MF NPs/FAM-labelled siRNA in hMSCs at different time points, and the nuclei were stained with DAPI (blue). (F) Quantification of the fluorescence intensity of (E). (G) Immunofluorescence stain for CH6-MF NPs/FAM-labelled siRNA (green) and lysosomes (red) by confocal laser scanning microscopy to track the intracellular fate of CH6-MF NPs, and the nuclei were stained with DAPI (blue). Data were analysed using one-way ANOVA. The outcomes are presented as the mean ± SD. ns = statistically nonsignificant, *P < 0.05, **P < 0.01, and ***P < 0.001; n = 3. (C) and (E) Scale bar = 200 μm. (G) Scale bar = 100 μm

For the future application of MF NPs and CH6-MF NPs, biosafety is the most important index. Here, the cytotoxicity was measured by CCK-8 assay. As shown in Fig. 3A and B, both MF NPs and CH6-MF NPs at concentrations up to 12 µg/mL showed negligible cytotoxicity to hMSCs after 72 h of incubation. Moreover, CH6-MF NPs slightly improved cell viability, which may be attributed to the PEG modification of CH6-MF NPs, as reported previously by Knop et al. [39] Thus, CH6-MF NPs at a final concentration of 10 µg/mL were selected for subsequent experiments. Next, to assess the targeting ability of the CH6-MF NPs, four typical cell lines, mouse RAW264.7 macrophages, human Chang liver cells, human marrow mesenchymal cells (hMSCs), and mouse MC3T3-E1 preosteoblasts, were used to evaluate cell uptake. To display the cellular uptake and distribution of nanoparticles intuitively, we used FAM-labelled siRNA to bind with nanoparticles and make the results visible. As shown in Fig. 3C, under the same treatment standard, the fluorescence intensity of FAM was very weak in RAW264.7 macrophages and Chang liver cells at all time points. In contrast, in the hMSCs and MC3T3-E1 preosteoblast groups, the FAM fluorescence signal was significantly stronger than that in the other two groups. Quantitative analysis of the FAM fluorescence intensity (Fig. 3D) showed that the mean fluorescence intensity in hMSCs and MC3T3-E1 cells was approximately 40 times stronger than that in Chang liver cells and more than 80 times stronger than that in RAW264.7 macrophages at 6 h, indicating the specific cell-selective targeting ability of hMSCs and MC3T3-E1 preosteoblasts after modification with the aptamer CH6. We further explored the differences in the cellular uptake of MF NPs and CH6-MF NPs by hMSCs. As expected, the fluorescence intensity of naked FAM-labelled siRNA was almost invisible at all time points, indicating that naked siRNA could not be absorbed by hMSCs. In contrast, when using MF NPs as vehicle, the fluorescence intensity in hMSCs increased with incubation time. Moreover, CH6-MF NPs/FAM-labelled siRNA could accumulate in hMSCs, with higher green fluorescence intensity than the group without CH6 modification, suggesting that modification of the aptamer CH6 led to the enhanced cellular uptake (Fig. 3E and F).

After the delivery of siRNA into hMSCs, various intracellular barriers must be overcome. Among these barriers, the escape of siRNA from endosomes or lysosomes into the cytoplasm is considered to be the most important [40]. We further tracked the intracellular fate of CH6-MF NPs by confocal laser scanning microscopy. Figure 3G shows that the fluorescent puncta of CH6-MF NPs/FAM-labelled siRNA were located at the edge of the cell contour after incubation for 1 h. At 3 h, the green fluorescent puncta showed clear colocalization with the red fluorescent puncta of endo/lysosomes stained with LysoTracker red. At 6 h, most of the green fluorescent puncta were separated from the red fluorescent puncta, which meant that the CH6-MF NPs/FAM-labelled siRNA successfully escaped from endo/lysosomes.

### Inhibition of abnormal osteogenic differentiation of hMSCs in Vitro

**Fig. 4:**
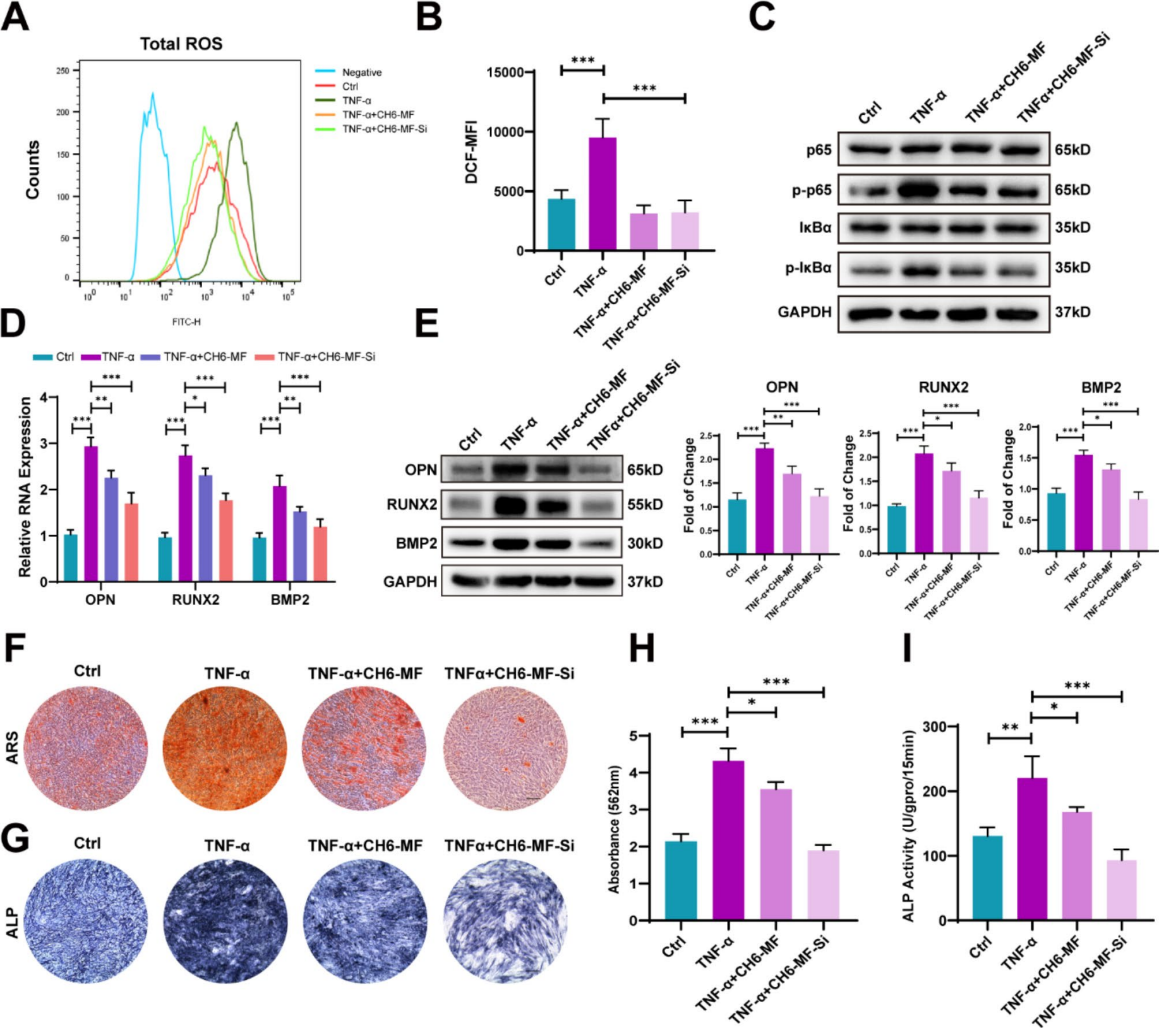
Anti-ROS effects and inhibition of abnormal osteogenic differentiation of hMSCs in vitro. (A) Cellular flow cytometry analysis of total ROS after treatment with PBS, TNF-α (10 ng/mL), CH6-MF NPs (10 µg/mL) + TNF-α and CH6-MF NPs/BMP2 siRNA nanocomplexes (CH6-MF-Si NPs, 10 µg/mL) + TNF-α for 48 h. (B) Quantitative analysis of ROS via detection of the mean fluorescence intensity (MFI) of DCF. (C) Western blot analysis of components of the nuclear factor kappa-B pathway on day 2 of PBS, TNF-α, TNF-α + CH6-MF NPs or TNF-α + CH6-MF-Si NPs stimulation. (D) Relative mRNA expression of OPN, RUNX2 and BMP2 was distinguished by qRT-PCR on day 12 of hMSC osteogenic differentiation after being treated with PBS, TNF-α, TNF-α + CH6-MF NPs or TNF-α + CH6-MF-Si NPs. (E) Western blot for OPN, RUNX2 and BMP2. The right panel shows the data quantification. (F) ARS staining of hMSCs treated with PBS, TNF-α, TNF-α + CH6-MF NPs or TNF-α + CH6-MF-Si NPs on day 12. (G) ALP staining of hMSCs treated with PBS, TNF-α, TNF-α + CH6-MF NPs or TNF-α + CH6-MF-Si NPs on day 12. (H) Quantitative analysis of ARS staining on day 12. (I) Quantitative analysis of ALP activity on day 12. Data were analysed using one-way ANOVA. The outcomes are presented as the mean ± SD. ns = statistically nonsignificant, *P < 0.05, **P < 0.01, and ***P < 0.001; n = 3. Scale bar = 100 μm

AS is a common rheumatic disorder distinguished by chronic inflammation and heterotopic ossification at local sites of entheses [6]. As an inflammatory factor, TNF-α triggers intracellular ROS production, which in turn cooperates with TNF-α to promote the development of inflammation [25, 26]. Here, we evaluated the ROS-scavenging effect of CH6-MF NPs in hMSCs using a DCFH-DA probe in vitro. After hMSCs were pretreated with CH6-MF NPs or CH6-MF NPs/BMP2 siRNA nanocomplexes (CH6-MF-Si NPs) for 6 h and then incubated with TNF-α for another 48 h, the intracellular ROS levels were evaluated through flow cytometry. Figure 4A–B illustrate that the ROS level was abnormally enhanced in the TNF-α-treated group more than in the control group. In contrast, the addition of CH6-MF NPs or CH6-MF-Si NPs markedly decreased the ROS level, which was consistent with Fig. 2.

We previously found that TNF-α promoted the proliferation, osteogenic differentiation and directional migration of hMSCs, which was also proven by Xie et al. [22], Li et al. [23] and Yang and Dai [24]. Moreover, the NF-κB signalling pathway is responsible for TNF-α-induced enhanced osteogenic differentiation in hMSCs [22, 41]. The IκBα protein, an important member of the inhibitor of kappa B (IκB) family of proteins, plays an inhibitory role in the NF-κB signalling pathway. When the NF-κB pathway is activated, IκBα is phosphorylated, and this phosphorylation triggers polyubiquitination and subsequent degradation of IκBα. NF-κB then translocates into the nucleus and interacts with phosphorylated p65, activating NF-κB target genes [42, 43]. Next, we evaluated the effect of CH6-MF NPs and CH6-MF-Si NPs on the NF-κB signalling pathway. After pretreatment with CH6-MF NPs and CH6-MF-Si NPs, the hMSCs were cultured for an additional 2 days in osteogenic medium with 10 ng/mL TNF-α to induce osteogenic differentiation. As shown in Fig. 4C, the protein expression levels of p-IκBα and p-p65 were increased in the TNF-α group. In contrast, p-IκBα and p-p65 decreased in both the CH6-MF NP group and CH6-MF-Si NP group, demonstrating that the NF-κB signalling pathway was excessively stimulated by TNF-α but blocked by CH6-MF NPs.

To assess the functional effects of CH6-MF NPs and CH6-MF-Si NPs on osteogenic differentiation under inflammatory conditions, we analysed the gene expression of osteoblastic markers, including BMP2, Runx2, and OPN. Osteogenesis-related gene expression levels were significantly enhanced in the TNF-α group, while the expression of these genes was decreased in the CH6-MF NP group, which may be related to blockade of the NF-κB pathway. Moreover, the decrease in gene expression levels in the CH6-MF-Si NP group was more significant than that in the CH6-MF NP group, confirming high knockdown efficiency (Fig. 4D). We then investigated the protein expression levels on day 12 during osteogenic differentiation. Similar to the above results, the protein expression levels increased in the TNF-α group more than those in the control group. Moreover, the abnormally enhanced osteoblastic protein expression levels were rescued by CH6-MF NPs and further decreased in the CH6-MF-Si NP group (Fig. 4E). Then, ALP production and extracellular matrix mineralization were measured. The TNF-α group had more intense ALP and ARS staining than the control, while the CH6-MF NPs reduced the ALP and ARS staining intensity. Significantly, the staining intensity of the CH6-MF-Si NP group was rescued to a level similar to that of the control group (Fig. 4F-H). The results of quantitative ALP assay (Fig. 4I) were consistent with the ALP staining results, illustrating that CH6-MF NPs could inhibit the abnormal osteogenic differentiation of hMSCs under inflammatory conditions and could nearly rescue this pathological change when loaded with BMP2 siRNA.

To our surprise, CH6-MF NPs promoted the osteogenesis of hMSCs in osteogenic differentiation medium without TNF-α (Figure S2A-C); this may be attributed to the reduction of intracellular ROS levels caused by CH6-MF NPs (Figure S3A and B), as a high level of ROS is known to suppress osteogenic differentiation [44, 45].

Taken together, these findings demonstrated that hMSCs under inflammatory conditions underwent abnormally strong osteogenic differentiation, while CH6-MF-Si NPs treatment markedly rescued this abnormal osteogenic differentiation.

### Therapeutic effect of CH6-MF NPs and CH6-MF-Si NPs in an in vivo model

**Fig. 5:**
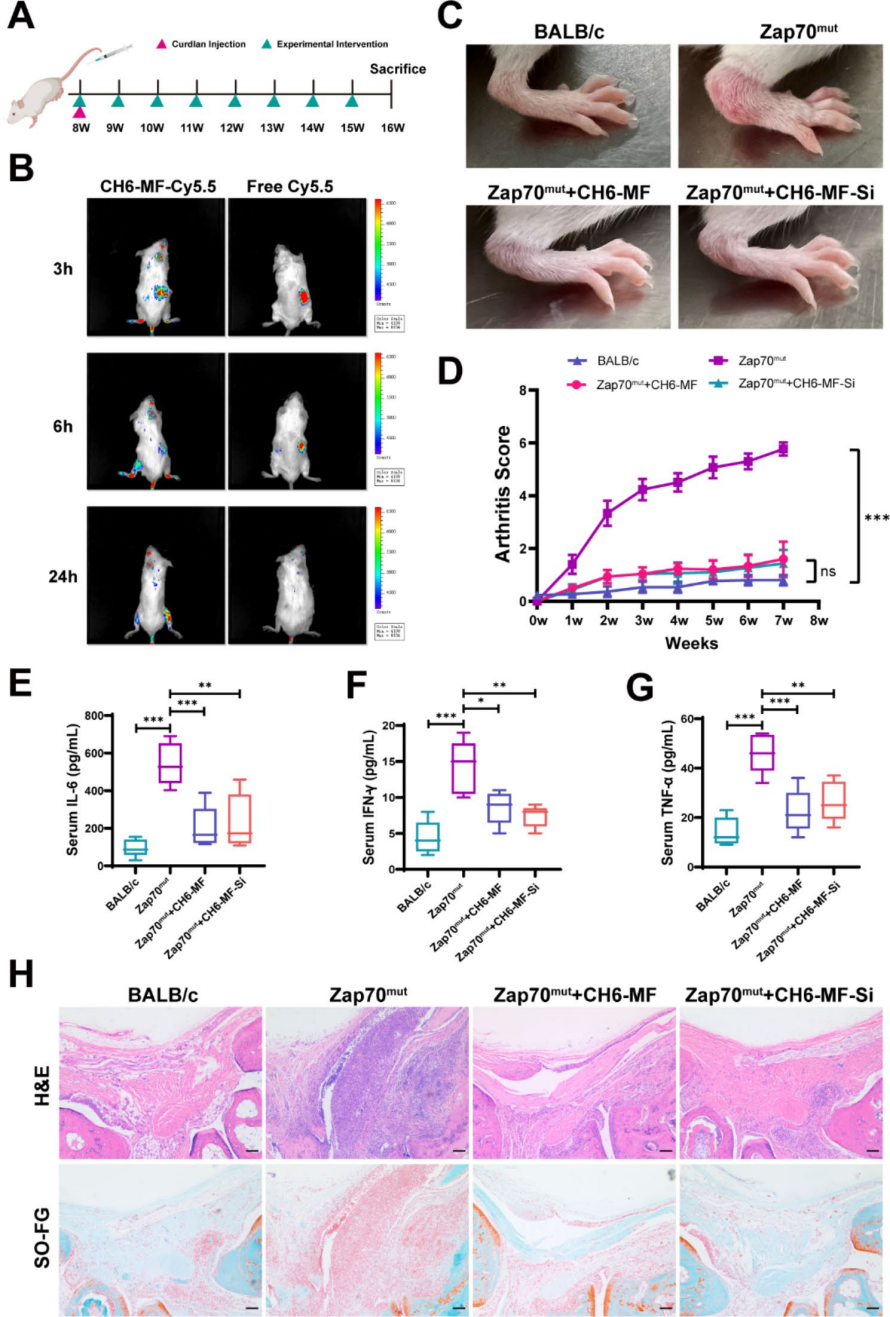
Therapeutic effect of CH6-MF NPs and CH6-MF-Si NPs in vivo. (A) The process used to test therapeutic effects in the in vivo model is shown in the schematic diagram. (B) In vivo imaging of CH6-MF-Cy5.5 and free Cy5.5 in Zap70^mut^ mice at different times. (C) Photographs of the hind paws of BALB/c or Zap70^mut^ mice treated with CH6-MF NPs or CH6-MF-Si NPs. (D) Inflamed joints in BALB/c or Zap70^mut^ mice treated with CH6-MF NPs or CH6-MF-Si NPs. The mice were evaluated with clinical scores. Average levels of inflammatory cytokines, including (E) interleukin-6 (IL-6), (F) interferon-γ (IFN-γ) and (G) tumour necrosis factor-α (TNF-α) were detected by ELISA. (H) Haematoxylin-eosin (H&E) and safranin-O (SO-FG) staining were used to assess inflammatory infiltration of the ankle joint. One-way ANOVA was performed on the data. The outcomes are presented as the mean ± SD. ns = statistically nonsignificant, *P < 0.05, **P < 0.01, and ***P < 0.001. Scale bar = 100 μm

To investigate the in vivo performance of CH6-MF NPs and CH6-MF-Si NPs for AS treatment in vivo, we further employed Zap70^mut^ mice to assess the treatment effects of CH6-MF NPs and CH6-MF-Si NPs on AS. At the time of curdlan induction, the mice were injected intravenously with CH6-MF NPs or CH6-MF-Si NPs, and then treatment was carried out once a week for 8 weeks (Fig. 5A). To evaluate the in vivo distribution of CH6-MF NPs, after intravenous administration of the Cy5.5-labelled CH6-MF NPs, the distribution of nanoparticles was investigated using a live imaging system. As shown in Fig. 5B, free Cy5.5 was rapidly cleared by the liver after 6 h in vivo. However, intense fluorescence of Cy5.5-labelled CH6-MF NPs was observed at the site of inflamed joints 3 h after administration and persisted for up to 24 h, suggesting the inflammation-targeting effect of CH6-MF NPs in vivo and their potential for continuous healing.

Previous studies have shown that curdlan-treated Zap70^mut^ mice could develop significant arthritis symptoms in peripheral and axial joints [46, 47]. The curdlan-treated Zap70^mut^ mice developed typical arthritis symptoms, eight weeks after the first immunization (Fig. 5C, S4A and B). We then measured the therapeutic effect of CH6-MF NPs and CH6-MF-Si NPs. The CH6-MF NP and CH6-MF-Si NP groups showed significant improvements in the redness and swelling of the ankle compared with the curdlan-treated group (Fig. 5C), and both groups exhibited significantly reduced clinical arthritis scores and better end outcomes than the curdlan-treated group (Fig. 5D). Since CH6-MF NPs were intravenously administered and accumulated at the site of inflammation, CH6-MF NPs could consistently scavenge ROS, exerting an antioxidative effect and anti-inflammatory effect during the process of circulation and accumulation. We further detected proinflammatory cytokine levels in the serum. As shown in Fig. 5E-G, the curdlan-treated Zap70^mut^ mice showed higher proinflammatory cytokine levels, such as TNF-α, IFN-γ, and IL-6, than the normal group, indicating the successful establishment of the AS model. Remarkably, CH6-MF NPs could reduce the levels of these proinflammatory cytokines to nearly normal levels, and the cytokine levels were also reduced with similar efficacy in the CH6-MF-Si NP group, demonstrating that both CH6-MF NPs and CH6-MF-Si NPs achieved good therapeutic efficacy by decreasing circulating proinflammatory cytokine levels. The anti-inflammatory effect of CH6-MF NPs and CH6-MF-Si NPs was further evaluated by histological analyses (Fig. 5H). Ankle joint sections were stained with haematoxylin-eosin (H&E) and safranin-O, and the joints in curdlan-treated Zap70^mut^ mice were seriously infiltrated by inflammatory cells. In contrast, significant improvement in symptoms was observed in both the CH6-MF NP and CH6-MF-Si NP groups, which showed minimal infiltration by inflammatory cells. These results suggested that CH6-MF NPs could exert good anti-inflammatory activity as expected, and the loaded BMP2 siRNA did not impact this anti-inflammatory effect.

**Fig. 6:**
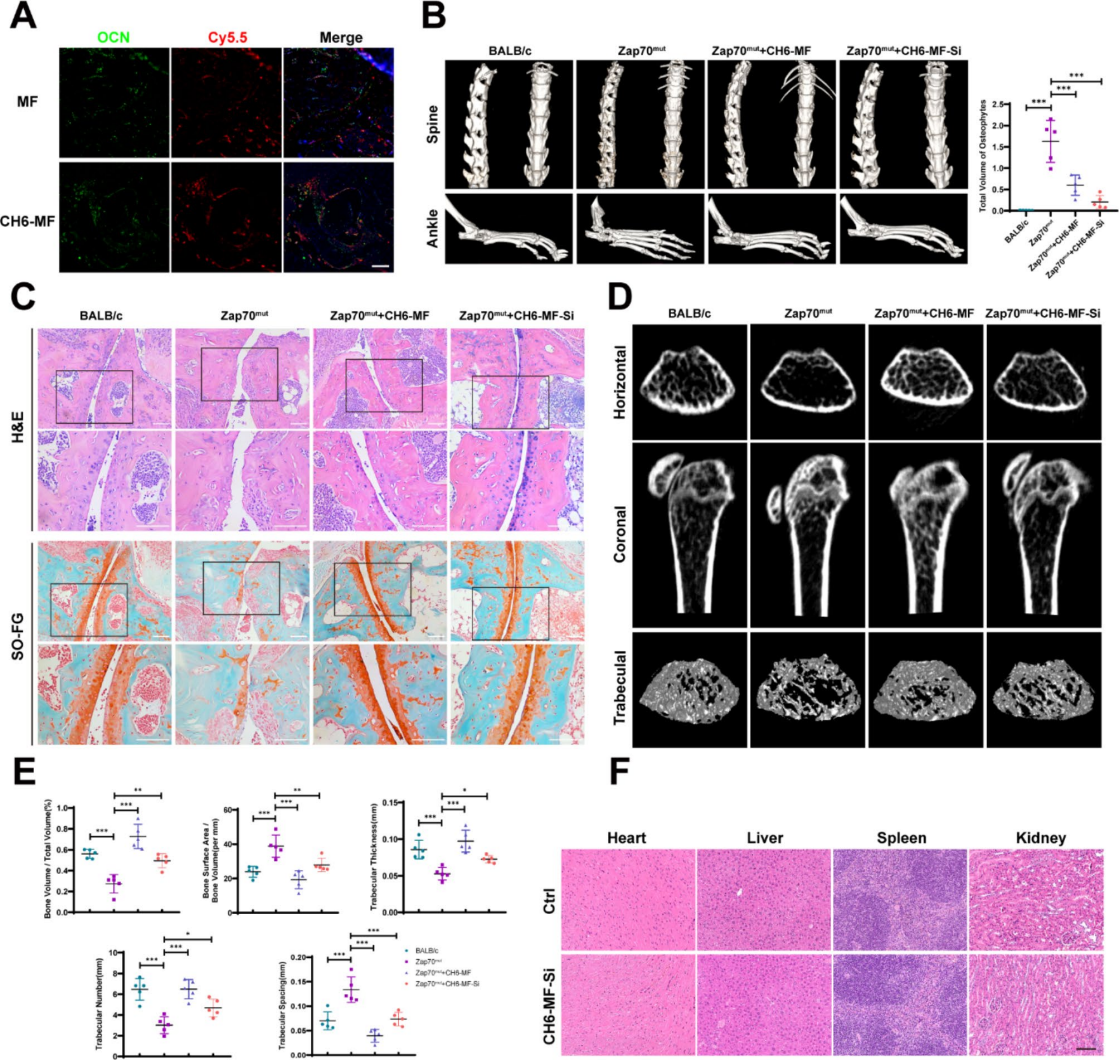
Inhibition of abnormal osteogenic differentiation in vivo. (A) Cell-selective targeting capacity of CH6-MF NPs in vivo. The effects were measured by using fluorescence micrographs of ankle sections. Colocalization of Cy5.5-labelled CH6-MF NPs (red) and OCN (green) was detected, and the nuclei were stained with DAPI (blue). (B) Three-dimensional micro-CT reconstruction of the spine and ankle. The results showed osteophyte formation in the spine and ankles, and the quantification of the total volume of osteophytes in ankles is shown in the right panel. (C) Haematoxylin-eosin (H&E) and Safranin-O staining (SO-FG) of ankle sections. The results showed cartilage damage and bone destruction of the ankle joint. (D) Micro-CT coronal and horizontal images of the proximal tibia. BALB/c or Zap70^mut^ mice were treated with CH6-MF or CH6-MF-Si NPs, and three-dimensional reconstruction was used to analyse the trabecular bone. (E) BA/BV, BV/TV, trabecular thickness, trabecular number and trabecular spacing. The results were analysed in BALB/c or Zap70^mut^ mice treated with CH6-MF or CH6-MF-Si NPs. (G) Haematoxylin-eosin (H&E) staining of the heart, liver spleen and kidney to show the safety of the nanoparticles. Data were analysed by one-way ANOVA. The outcomes are presented as the mean ± SD. ns = statistically nonsignificant, *P < 0.05, **P < 0.01, and ***P < 0.001. Scale bar = 100 μm

Next, the cell-selective targeting capacity and the effect on abnormal new bone formation of CH6-MF NPs and CH6-MF-Si NPs were investigated in vivo. First, to investigate whether CH6-MF NPs could be taken up in an osteoblast-specific manner in vivo, the colocalization of Cy5.5-labelled nanoparticles and osteocalcin (OCN), a marker of osteoblasts, in ankle sections was measured. As shown in Fig. 6A, Cy5.5-labelled CH6-MF NPs and OCN^+^ cells were strongly colocalized in vivo. In contrast, no obvious overlapping staining was observed with Cy5.5-labelled MF NPs, suggesting a good cell-selective targeting capacity of CH6-MF NPs in vivo.

Heterotopic ossification at local entheses sites coexists with bone destruction and systemic osteoporosis in both AS patients and mouse models [48–52]. In the very early stage of AS, bone marrow oedema usually precedes imaging findings of joint destruction [53]. Then, erosive changes, including cartilage destruction and bone erosion, are involved in the development of pathology [54]. Moreover, during the chronic stage, chronic inflammatory exposure leads to abnormally enhanced skeletal anabolism at the inflammation site, which may aggravate the resorption of trabecular bone even further [55, 56]. Given the anti-ROS and anti-inflammatory activities of CH6-MF NPs, we speculate that the nanoparticles may exert a protective effect against this abnormal bone mass imbalance in AS. As shown in Fig. 6B, three-dimensional (3D) reconstruction by micro-CT showed that the symptoms of AS in both the spine and ankles were abnormal osteophyte formation and destruction of facet joints in the Zap70^mut^ model. CH6-MF NPs with or without siRNA can alleviate the bone destruction of facet joints in both the spine and ankles. When loaded with BMP2 siRNA, CH6-MF-Si NPs further alleviated the abnormal heterotopic ossification at local sites of entheses in the ankles of Zap70^mut^ mice. Similar results were observed in ankle joint sections stained with haematoxylin-eosin (H&E) and safranin-O (Fig. 6C). The curdlan-treated group showed almost complete destruction of cartilage and severe bone erosion. Importantly, compared with the saline-treated Zap70^mut^ mice, obvious improvements in these symptoms were observed in both the CH6-MF NP and CH6-MF-Si NP groups, with smooth and complete cartilage surfaces as well as minimal bone destruction. The bone mass of the upper tibia was further analysed with CT images and 3D reconstruction images (Fig. 6D), and the BALB/c and Zap70^mut^ groups revealed that the local inflammation of Zap70^mut^ mice resulted in bone loss and reduction of trabeculae. The bone mass was significantly elevated in the group treated with CH6-MF NPs more than in the Zap70^mut^ group because of the anti-inflammatory effects of the CH6-MF NPs. The bone mass of the CH6-MF-Si NP group was close to that of BALb/c mice, which was higher than that of the inflammatory mouse model. Furthermore, trabecular bone indicators (BV/TV, BA/BV, trabecular thickness, trabecular number, and trabecular spacing) were analysed (Fig. 6E), and the results of trabecular analysis were consistent with the outcomes of CT images and 3D reconstruction images of trabeculae.

In summary, these results confirmed that CH6-MF NPs can relieve the abnormal bone mass imbalance observed in AS. When loaded with BMP2 siRNA, CH6-MF-Si NPs further relieved heterotopic ossification at local sites of entheses. However, there still remain some limitations in this study. For instance, the pathological mechanisms of heterotopic ossification in AS are complex, and the enhanced expression level of BMP2 is one of the potential mechanisms of abnormal new bone formation in AS. The inhibition of BMP2 expression alone may not be enough for the treatment of heterotopic ossification in AS. Further investigation is required to find more extensive and effective targets for the treatment of abnormal new bone formation in AS.

Finally, as CH6-MF-Si NPs were administered systemically, we then explored the safety of nanoparticles for AS therapy by histopathological analysis (Fig. 6F). No obvious changes or inflammation were detected in organ sections of the heart, liver, spleen and kidney by haematoxylin-eosin (H&E) staining. In short, all these outcomes indicated that CH6-MF NPs could be used as an effective and safe cell-selective drug carrier platform for AS therapy and highlighted the therapeutic potential of CH6-MF NPs/BMP2 siRNA for dual treatment of both chronic inflammation and heterotopic ossification in AS.

## Conclusion

In summary, we developed the CH6-MF NPs/siRNA nanosystem as an anti-ROS osteoblast-specific delivery system that can efficiently scavenge ROS and reduce abnormal inflammatory osteogenesis in vivo. When loaded with BMP2 siRNA, the nanoparticles (CH6-MF-Si NPs) exerted a good therapeutic effect on both chronic inflammation and heterotopic ossification of AS. This study indicates that CH6-MF NPs can be used efficiently as an alleviator of inflammation and as an osteoblast-specific delivery system and highlights the potential of CH6-MF NPs/BMP2 siRNA for the dual treatment of both chronic inflammation and heterotopic ossification in AS.

## Materials and methods

### Synthesis and characterization of CH6-MF NPs

The fabrication of MnFe_2_O_4_ was conducted based on a previous study [57] with slight modification. Briefly, 0.02 mol MnCl_2_ (2.5 g) and 0.03 mol FeCl_2_ (3.8 g) were dispersed in 100 mL ddH2O with constant stirring, and the solution was heated to 70 °C and protected by N_2_. Subsequently, 70 mL 6 M NaOH was applied to the above mixture and reacted at 70 °C for 1.5 h, and the product MnFe_2_O_4_ was centrifuged (8000 rpm, 10 min) and washed with water several times to remove excess NaOH. For the fabrication of PEI-capped MnFe_2_O_4_ NPs (MF NPs), 100 mg MnFe_2_O_4_ was dispersed in 20 mL of ddH_2_O, and then 0.2 g PEI (mw = 25 kDa) was applied to the solution with agitation at room temperature for 24 h. The particles were exposed to three rounds of washing with water to remove excess PEI. For the fabrication of CH6-MF NPs, the osteoblast-specific aptamer CH6 was designed and modified according to a previous report [37]. Ten nanomolar NHS-PEG-Mal, 5 nM CH6-SH and 5 mg/mL MF NPs were mixed and agitated overnight at 4 °C protected from light, and the product was washed with water 3 times.

The morphologies of the nanoparticles were characterized by transmission electron microscopy (TEM), and the interplanar spacing was further measured by high-resolution transmission electron microscopy (HRTEM). X-ray diffraction (XRD) spectra were obtained to verify the crystallinity of the nanoparticles, and nitrogen adsorption-desorption isotherms were employed to study the porous structure. UV‒vis spectra of the nanoparticles were generated to verify the PEI modification on the MnFe_2_O_4_ surface using a UV spectrophotometer. To demonstrate successful modification with the aptamer CH6, we used Fourier transform infrared spectroscopy (FTIR) and UV‒Vis spectrophotometry.

### siRNA binding of CH6-MF NPs

The MF NPs and CH6-MF NPs were diluted with saline and then mixed with siRNA for 20 min. To confirm their siRNA binding ability, we employed agarose gel electrophoresis to examine mixtures with different NP/siRNA weight ratios (w/w) ranging from 5 to 30. The surface zeta potential of the CH6-MF NPs was also measured to confirm their binding ability.

### Serum stability assay

The MF NPs and CH6-MF NPs were diluted with saline and complexed with siRNA at a NP:siRNA weight ratio of 10:1 for 20 min. The complexes were incubated with fetal bovine serum (FBS) at a final concentration of 50% at 37 °C for different times. After incubation, samples were mixed with 50 µg of heparin (heparin:siRNA weight ratio of 100:1) for 30 min at RT. After centrifugation (12,000 rpm, 15 min, 4 °C), the supernatants were analysed by agarose gel electrophoresis(110 V, 5 min).

### Catalase-like activity of CH6-MF NPs

H_2_O_2_**(**2 mM) and standard MnFe_2_O_4_, MF NPs or CH6-MF NPs at a concentration of 10 µg/mL were mixed at room temperature. CAT kit (Nanjing Jiancheng, China) was employed to measure the H_2_O_2_ concentration every 50 s for 5 min. The enzymatic reaction rates of standard MnFe_2_O_4_, MF NPs and CH6-MF NPs (10 µg/mL) were investigated at different concentrations of H_2_O_2_ (8, 4, 2, 1, and 0.5 mM). The enzymatic kinetic parameters were determined with the Michaelis − Menten equation and the Lineweaver − Burk double reciprocal curve. The valence states of Mn and Fe in MF NPs were identified by X-ray photoelectron spectroscopy (XPS).

### Peroxide (POD)-Like activity of CH6-MF NPs

3,3,5,5-Tetramethylbenzidine (TMB) was used to investigate the POD-like activity of the NPs. TMB (0.5 mM), H_2_O_2_ (1 mM) and different nanoparticles (standard MnFe_2_O_4_, MF NPs, and CH6-MF NPs at a concentration of 10 µg/mL) were mixed individually with a 0.1 M acetate buffer solution (NaAc, pH = 4.5) at room temperature. Then, the intensity of the developed colour (as indicated by the absorbance at 650 nm for TMB) was recorded every 50 s for 5 min to investigate POD-like activity.

### Superoxide dismutase (SOD) activity of CH6-MF NPs

The SOD-like activity of the NPs was studied by formazan formation using a (2-(4-iodophenyl)-3-(4-nitrophenyl)-5-(2,4-disulfophenyl)-2 H-tetrazolium (WST-1) assay kit (Nanjing Jiancheng Bioengineering Institute, China). Briefly, WST working solution and enzyme working solution were sufficiently mixed with 10 µg/mL standard MnFe_2_O_4_, MF NPs or CH6-MF NPs at room temperature for 20 min. Then, we used a microplate reader to evaluate the absorbance of the developed colour at 450 nm and the formazan concentration, and the inhibition rate was used to present the SOD activity (%).

### Cell isolation and culture

This study was approved by the ethics committee of the Eighth Affiliated Hospital, Sun Yat-Sen University, Guangzhou, China. hMSCs were isolated and cultured by our previously reported methods [58]. hMSCs at passages 3–5 were used in subsequent trials. hMSCs, mouse MC3T3-E1 preosteoblasts, mouse RAW264.7 macrophages and human Chang liver cells were seeded in culture flasks and cultured in Dulbecco’s modified Eagle’s medium (DMEM; Gibco, New York, USA) with 10% FBS at 37 °C and 5% CO_2_. Upon reaching ~ 90% confluence, the cells were digested and then passaged and used for further experiments.

### Cell viability

hMSCs were seeded in 96-well plates at a density of 3 × 10^3^ cells per well. After the cells were cultured overnight, MF NPs and CH6-MF NPs were added to the medium at different concentrations (0, 2, 4, 6, 8, 10, and 12 µg/mL) and incubated for another three days. Then, the cell viability was measured by a Cell Counting Kit-8 (Beyotime, China) according to the manufacturer’s protocol.

### Cell uptake assays and cell-selective delivery in vitro

hMSCs, MC3T3-E1 preosteoblasts, RAW264.7 macrophages and Chang liver cells were used to investigate cell-selective delivery in vitro. Cells (0.5 × 10^5^) were seeded and cultured overnight in the wells of 12-well plates. Then, MF NPs or CH6-MF NPs premixed with FAM-labelled siRNA were applied to the culture and inserted into the incubator for 1, 3, and 6 h. After treatment, the cells were washed three times with PBS. The cell-selective delivery capacity of CH6-MF NPs was evaluated by using a fluorescence microscope.

To investigate subcellular localization, 0.8 × 10^5^ hMSCs were seeded in a 35 mm confocal dish and cultured overnight. Then, the CH6-MF NPs premixed with FAM-labelled siRNA were added to the medium. After 1, 3, or 6 h of incubation, the cells were exposed to three rounds of washing with PBS then incubated with Lysotracker Red (Beyotime, China) to label endo/lysosomes according to the manufacturer’s recommendation. Then, the cells were fixed for 20 min, DAPI (1 µg/mL) was used to stain nuclei, and the intracellular distribution of CH6-MF NPs was obtained by a confocal laser scanning microscope (LSM880).

### Intracellular ROS assay

We used the Reactive Oxygen Species Assay Kit (Beyotime, China) for investigating intracellular ROS levels according to the manufacturer’s protocol. Briefly, after hMSCs were preincubated with 10 µg/mL CH6-MF NPs or CH6-MF NPs/BMP2 siRNA nanocomplexes (CH6-MF-Si NPs, w/w ratio 10:1) for 6 h and washed 3 times to remove free nanoparticles, TNF-α at a final concentration of 10 ng/mL was applied to the culture. After another 48 h, the hMSCs were exposed to three rounds of washing with PBS then incubated with DCFH-DA for 20 min at 37 °C. Then, the cells were collected, and the fluorescence and mean fluorescence intensity (MFI) of DCF were measured with a flow cytometer (BD Influx, BD Bioscience) to evaluate intracellular ROS levels.

### RNA extraction, reverse transcription, and real-time PCR

hMSCs (0.5 × 10^5)^ were seeded and cultured in the wells of 12-well cell culture plates overnight at 37 °C with 5% CO_2_. hMSCs were then incubated with 10 µg/mL CH6-MF NPs or CH6-MF-Si NPs for 6 h and washed three times to remove the extracellular nanocomplexes. Then, the hMSCs were cultured for an additional 12 days in osteogenic differentiation medium (DMEM with 10% FBS, 100 IU/ml penicillin, 100 IU/ml streptomycin, 50 mM ascorbic acid, 10 mM b-glycerol phosphate, and 0.1 mM dexamethasone) to induce osteogenic differentiation. TNF-α (10 ng/mL) was used in the above osteogenic differentiation medium to mimic the abnormal osteogenic differentiation of hMSCs under inflammatory conditions. Then, we used TRIzol to isolate total RNA from hMSCs and used a PrimeScript^™^ RT reagent kit to transcribe RNA into cDNA. We used the LightCycler® 480 PCR System (Roche) to carry out the real-time PCR assay, measuring the gene expression of BMP2, Runx2, and OPN to investigate the effects of CH6-MF NPs on osteogenic differentiation and the knockdown efficiency of CH6-MF-Si NPs. The primer, human BMP2 siRNA and control siRNA sequences are available in Tables S1-3, and the 2^−ΔΔCt^ method was employed to evaluate relative gene expression levels.

### Protein extraction and western blotting

Protein from lysed cells was separated by centrifugation (12,000 rpm, 30 min at 4 °C). After the protein was quantified and denatured by boiling, equal concentrations of protein were loaded on sodium dodecyl sulfate-polyacrylamide gels for electrophoresis, and then the separated proteins were transferred to PVDF membranes (Millipore). The PVDF membranes were blocked by using skim milk, followed by incubation for ~ 12 h at 4 °C with primary antibodies against BMP2, RUNX2, OPN, p-p65, p65, IKBα, p-IKBα, and GAPDH (1:1000). Then, the PVDF membranes were incubated with a horseradish peroxidase (HRP)-conjugated secondary antibody (1:3000) at room temperature for an hour. After washing three times, specific antibody-antigen complexes were determined by Immobilon Western Chemiluminescent HRP Substrate.

### Alkaline phosphatase (ALP) assay and ARS staining

For the quantitative ALP activity assay, equal amounts of protein extracted from hMSCs were inserted into the incubator with reaction buffer (Nanjin Jiancheng Biotech, China, A059-2). The reaction was allowed to proceed sufficiently at 37 °C for 15 min, then stopped, and the absorbance at 520 nm was measured to evaluate ALP activity. ALP activity was defined as units per gram protein (U/gprot/15 min). For the qualitative ALP activity assay, hMSCs were treated with a fixative solution composed of citrate-acetone-formaldehyde and inserted into the incubator for 15 min with alkaline dye solution to acquire images. Then, the samples were examined and photographed with a microscope.

For ARS staining, hMSCs were treated with 4% fixative solution and incubated with 1% Alizarin red (ARS, pH 4.3) solution for 15 min. The stained cells were washed three times, observed and photographed under a microscope.

### Generation of the Zap70-W163C mutant mouse model (Zap70^mut^), mouse induction, treatment and therapeutic efficacy

Mice with the W163C point mutation of murine Zap70 were purchased and used as an arthritis mouse model, as reported previously [47, 59]. Genetically engineered mice were designed by Shanghai Model Organisms Center, Inc. (Shanghai, China). The CRISPR/Cas9 gene-editing technique was used to establish an arthritis mouse model. Briefly, transcription of Cas9 mRNA was carried out in vitro by using the mMESSAGE mMACHINE T7 Ultra Kit (Ambion, TX, USA), and then we carried out purification with the MEGAclearTM Kit (ThermoFisher, USA). The selected Cas9-targeted guide RNA (sgRNA), an RNA with the sequence 5’-CAGCCCACGAGCGAATGCCCTGG-3’, was transcribed and purified in vitro. To produce the F0 point mutant mice, the fertilized eggs of BALB/c mice were coinjected with transcribed Cas9 mRNA and sgRNA as well as a 161 base pair single-stranded oligodeoxynucleotide (ssODN). After identification by PCR and sequencing (primer pairs: F1: 5’-CCTCCCTGGGTGGATTAGGA-3’; R1: 5’-AATGCAGGTGACTCCAGCTC-3’), the expected F0 point mutant mice were selected for hybridization with BALB/c mice. The genotype of F1 mice was confirmed by PCR and sequencing. The sequence of the ssODN for generating point mutant mice was 5’-GGCGATGCACTAGAGCAGGCCATCATCAGCCAGGCCCCACAGGTGGAGAAGCTCATTGCTACCACAGCCCACGAGCGAATGCCCTGCTATCACAGCAGCCTGACTCGTGAGGAGGCCGAGCGCAAACTCTATTCCGGCCAGCAGACCGACGGCAAGTTCCT-3’. The animal experimental plan was approved by the Animal Ethical and Welfare Committee of Sun Yat-sen University (SYSU-IACUC-2022-001078). Following the guidelines of the Institutional Animal Care and Use Committee of Sun Yat-Sen University, we used male mice in this study. Immunization was induced at 8 weeks of age by intraperitoneal injection of 3 mg curdlan. The Zap70^mut^ mice were separated into three groups in a random manner (n = 5): a saline group, a CH6-MF NP (CH6-MF) group, and a CH6-MF NP/BMP2 siRNA (CH6-MF-Si) group. Moreover, normal healthy mice with no treatment were employed as a negative control for symptom comparison with the arthritis mouse model. Intravenous administration of different formulations started at the time of immunization, and the mice were administered consecutively every week for a total of eight times. The dose of CH6-MF NPs and CH6-MF-Si NPs was 10 µg/g BW. Clinical symptoms were assessed weekly by three blinded independent observers, starting from the time of immunization. The clinical arthritis score criteria were described previously: [46] 0 = no swelling or redness, 0.1 = swelling or redness of the digits, 0.5 = mild swelling and/or redness of the wrist or ankle joints, and 1 = severe swelling of the larger joints. The mice were sacrificed after eight consecutive administrations. Blood was collected, and the levels of TNF-α, IL-6 and IFN-γ were determined by ELISA (Dogesce, China) according to the manufacturer’s instructions. The serum levels of MDA were measured via a MDA assay kit (Nanjing Jiancheng, China), and the levels of AOPP were determined by an AOPP ELISA kit (Boyun Biotech, Shanghai, China) according to the manufacturer’s protocols. The serum levels of BMP2 were measured using a mouse BMP2 ELISA kit (Cohesion Biosciences). Micro-CT scanning, haematoxylin and eosin (H&E) staining, immunofluorescence, and safranin-O/fast green staining were carried out with harvested tissues. Mouse BMP2 siRNA sequences are available in Table S4.

### Biodistribution of CH6-MF NPs in an in vivo model

Four weeks after immunization, Cy5.5-labelled CH6-MF NPs and free Cy5.5 were administered via intravenous injection in Zap70^mut^ mice. At 3, 6 and 24 h after injection, intraperitoneal administration of 4% (W/V) chloral hydrate was administered intraperitoneally to anaesthetize the mice. The in vivo biodistribution of nanoparticles was visualized by the fluorescence of Cy5.5 using the Xenogen IVIS Spectrum system (Calliper Life Sciences, Inc.).

### Micro-CT scanning

Micro-CT scanning was used to visualize and analyse the bone quality of the spine, proximal tibia, and ankle. Tissues from sacrificed mice were treated with formaldehyde fixative solution for 2 days and then scanned and analysed using a micro-CT system (Siemens). Briefly, the proximal tibia, ankle and lumbar tissues were scanned, and then two- and three-dimensional structures were reconstructed. For the proximal tibia tissues, the region of interest (ROI) was defined as the region beneath the lower growth plate (approximately 50 mm and 100 slices). Bone morphometry was performed by measuring the bone volume/total volume (BV/TV), bone surface area/bone volume (BA/BV), trabecular thickness (Tb.Th), trabecular number (Tb.N) and trabecular spacing (Tb.Sp). For the ankle tissues, the region of new bone formation in front of the ankle was selected and analysed by calculating the volume of osteophytes.

### Cell-selective delivery in vivo

The Zap70^mut^ mice were injected intravenously with Cy5.5-labelled CH6-MF NPs or Cy5.5-labelled MF NPs 12 h before the mice were sacrificed. Then, proximal tibia and ankle tissues from the Zap70^mut^ mice were fixed with formaldehyde fixative solution for 2 days, and decalcification of tissues was carried out by using 10% EDTA solution for one month. After dehydration, tissues were embedded in paraffin, and 4 µM sections were prepared for staining. The efficiency of cell-selective delivery was evaluated by histological analysis. To confirm the cell-selective targeting capacity of CH6-MF NPs, an anti-OCN monoclonal antibody (Abcam, USA) was used to label osteoblasts, and goat anti-mouse H&L fluorophore-labelled antibodies were used as secondary antibodies.

### H&E staining and safranin O staining

Harvested heart, kidney, liver, spleen, and ankle tissue samples from Zap70^mut^ mice and normal healthy mice were fixed with formaldehyde fixative solution for 2 days, and decalcification of ankle tissues was carried out by using 10% EDTA solution for one month. After embedding in paraffin, tissue samples were exposed for sectioning and stained with haematoxylin solution for 10 min. The sections were washed with water for 10 min, and eosin staining was carried out for 3 min. Then, the dehydrated sections were visualized and photographed by using a microscope. Safranin-O/fast green staining was carried out to evaluate the destruction of hard tissue. The ankle tissue samples from the Zap70^mut^ mice were fixed, decalcified, embedded and sectioned as described above and then stained with 0.02% of Fast Green FCF and 0.1% of safranin O. Then, the sections were visualized and photographed under a microscope.

### Safety evaluation in vivo

The healthy BALB/c mice at 8 weeks of age were administered intravenously with CH6-MF-Si NPs (10 µg/g BW) every week for overall 8 times. After the final administration, the mice were sacrificed and the heart, kidney, liver, and spleen tissue samples were collected for H&E staining and histological analysis.

### Statistical analysis

Statistical analysis was carried out by SPSS 26.0 and GraphPad Prism 8. The data are presented as the mean ± standard deviation (SD). Independent-sample t tests were employed to analyse significant differences between two experimental groups and one-way ANOVA with Bonferroni’s test was employed to analyse the differences among three or more groups. Values of * p < 0.05, **p < 0.01, and ***p < 0.001 were considered statistically significant.

## Electronic supplementary material

Below is the link to the electronic supplementary material.

Supplementary Material 1

## Data Availability

The data supporting the results of this investigation are available upon reasonable request from the corresponding author.

## Abbreviations

AS: ankylosing spondylitis
NSAIDs: nonsteroidal anti-inflammatory drugs
DMARDs: disease-modifying anti-rheumatic drugs
TNF: tumor necrosis factor
PEI: polyethylenimine
MF NPs: PEI-capped MnFe2O4 NPs
CH6-MF NPs: MF NPs modified by the aptamer CH6
ROS: reactive oxygen species
siRNA: Small interfering RNA
CH6-MF-Si NPs: CH6-MF NPs loaded with BMP2 siRNA
NF-ƙB: nuclear factor kappa-B
hMSCs: human mesenchymal stem cells
MSCs: mesenchymal stem cells
BMP2: bone morphogenic protein-2
HRTEM: high resolution transmission electron microscope
XRD: X-ray diffraction
FTIR: fourier transform infrared spectroscopy
PBS: phosphate-buffered saline
XPS: X-ray photoelectron spectroscopy
Km: Michaelis − Menten constant
Vma: maximum reaction rate
POD: peroxidase
SOD: superoxide dismutase
TMB: 3,3,5,5-Tetramethylbenzidine
CCK-8: Cell Counting Kit-8
PEG: polyethylene glycol
DAPI: 4,6-diamidino-2-phenylindole
FAM: 5-carboxyfluorescein
OPN: osteopontin
MFI: mean fluorescence intensity
RUNX2: Runt-related transcription factor 2
DCFH-DA: 2,7-Dichlorodihydrofluorescein diacetate
ALP: alkaline phosphatase
ARS: Alizarin Red S
Zap70mut: Zap70-W163C mutant mouse model
Cy5.5: Cyanine5.5
H&E: Haematoxylin-eosin
SO-FG: safranin-O/fast green
OCN: osteocalcin
micro-CT: micro computed tomography
BV/TV: bone volume/total volume
BA/BV: bone surface area/bone volume
Tb.Th: trabecular thickness
Tb.N: trabecular number
Tb.Sp: trabecular spacing
TEM: transmission electron microscopy
WST-1: (2-(4-iodophenyl)-3-(4-nitrophenyl)-5-(2,4-disulfophenyl)-2H-tetrazolium
DMEM: Dulbecco’s modified Eagle’s medium
RT–PCR: Real-time quantitative reverse transcription-polymerase chain reaction
TBST: Tris-buffered saline-Tween
PVDF: Poly Vinyli Dene Fluoride
HRP: horseradish peroxidase
sgRNA: small guide RNA
mRNA: messenger RNA
ssODN: single-stranded oligodeoxynucleotide
ELISA: Enzyme-linked Immunosorbent Assay
ROI: region of interest
EDTA: Ethylene Diamine Tetraacetic Acid
