# Supplementary material for: A novel Anti-ROS osteoblast-specific delivery system for ankylosing spondylitis treatment via suppression of both inflammation and pathological new bone formation
Source: J Nanobiotechnology. 2023 May 26;21:168. doi: 10.1186/s12951-023-01906-2 (PMC10214592; doi:10.1186/s12951-023-01906-2)
Supplement: Supplementary file 1 — Supplementary Material 1 [file 12951_2023_1906_MOESM1_ESM.docx]

| **Supplemental Table S1. Primer sequences for real-time PCR** | | | | |
| --- | --- | --- | --- | --- |
| Primer sequence | | | | |
| OPN for human | | Forward |  | 5’- CTCCATTGACTCGAACGACTC -3’ |
|  |  | Reverse |  | 5’- CAGGTCTGCGAAACTTCTTAGAT -3’ |
| RUNX2 for human | | Forward |  | 5’- TGGTTACTGTCATGGCGGGTA -3’ |
|  |  | Reverse |  | 5’- TCTCAGATCGTTGAACCTTGCTA -3’ |
| BMP2 for human | | Forward |  | 5'- ACCCGCTGTCTTCTAGCGT -3' |
|  |  | Reverse |  | 5'- TTTCAGGCCGAACATGCTGAG -3' |
| GAPDH for human | | Forward |  | 5’- GGAGCGAGATCCCTCCAAAAT -3’ |
|  |  | Reverse |  | 5’- GGCTGTTGTCATACTTCTCATGG -3’ |

Abbreviations: *OPN*, osteopontin; *RUNX2*, RUNX family transcription factor 2; *BMP2*, bone morphogenetic protein 2; GAPDH, glyceraldehyde-3-phosphate dehydrogenase.

| **Supplemental Table S2. siRNA sequences for BMP2 of human** | | | | | |
| --- | --- | --- | --- | --- | --- |
| siRNA sequence | | | | | |
| h-BMP2-siRNA | |  | sense |  | 5′-CAAGAGACAUGUUAGGAUATT-3′ |
|  |  |  | antisense |  | 5′-UAUCCUAACAUGUCUCUUGTT-3′ |

| **Supplemental Table S3. siRNA sequences for control** | | | | | |
| --- | --- | --- | --- | --- | --- |
| siRNA sequence | | | | | |
| NC-siRNA | |  | sense |  | 5′-CCGAAAGGCUGGAUACCUUTT-3′ |
|  |  |  | antisense |  | 5′-AAGGAUCCUGAACUGCUGGTT-3′ |

| **Supplemental Table S4. siRNA sequences for BMP2 of mouse** | | | | | |
| --- | --- | --- | --- | --- | --- |
| siRNA sequence | | | | | |
| m-BMP2-siRNA | |  | sense |  | 5′-GCAUCUUGUUCUUUCUUAATT-3′ |
|  |  |  | antisense |  | 5′-UUAAGAAAGAACAAGAUGCTT-3′ |


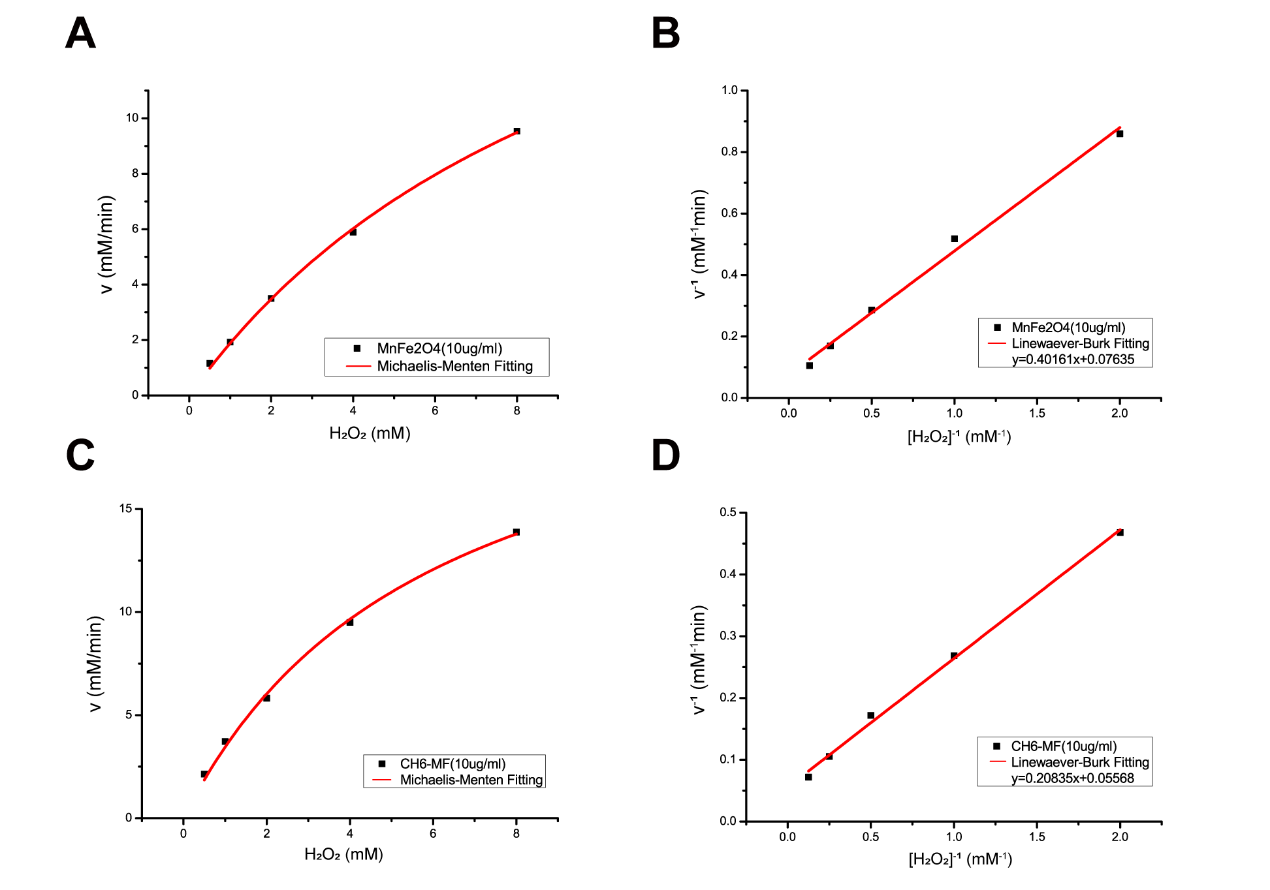


Figure S1. Michaelis−Menten kinetic analysis and Lineweaver−Burk plot of the CAT-like activity of commercial MnFe_2_O_4_ (A and B) and CH6-MF NPs (C and D) with H_2_O_2_ as the substrate at 25 °C.


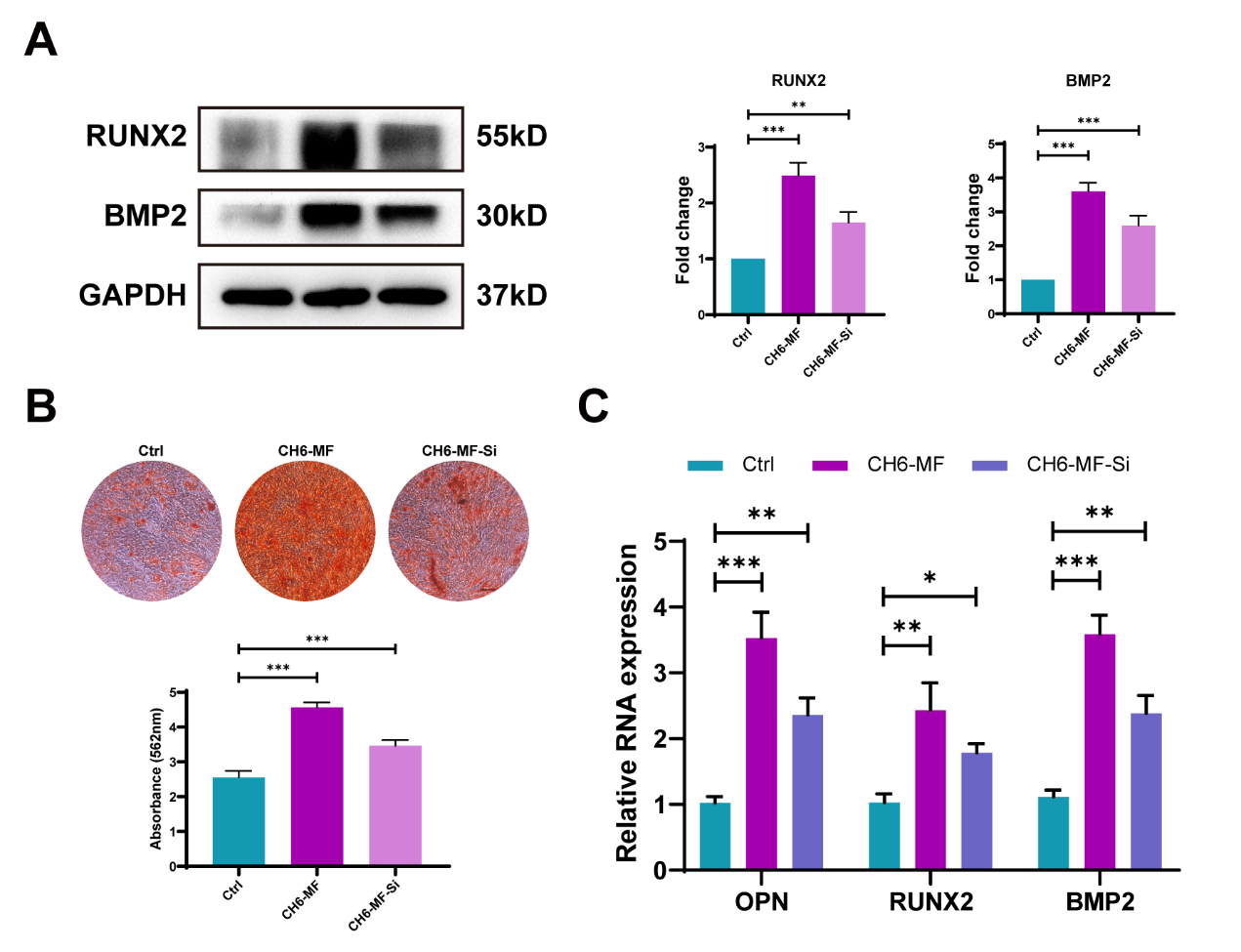


Figure S2. Promotion of NPs on osteogenic differentiation of hMSCs without TNF-α in vitro. (A) Western blot for RUNX2 and BMP2. Quantification of the data is shown in the right panel. (B) ARS staining of hMSCs treated with PBS, CH6-MF and CH6-MF-Si NPs on day 12. (C) Relative mRNA expression of OPN, RUNX2 and BMP2 on day 12 of hMSC osteogenic differentiation after PBS, CH6-MF and CH6-MF-Si NPs stimulation.


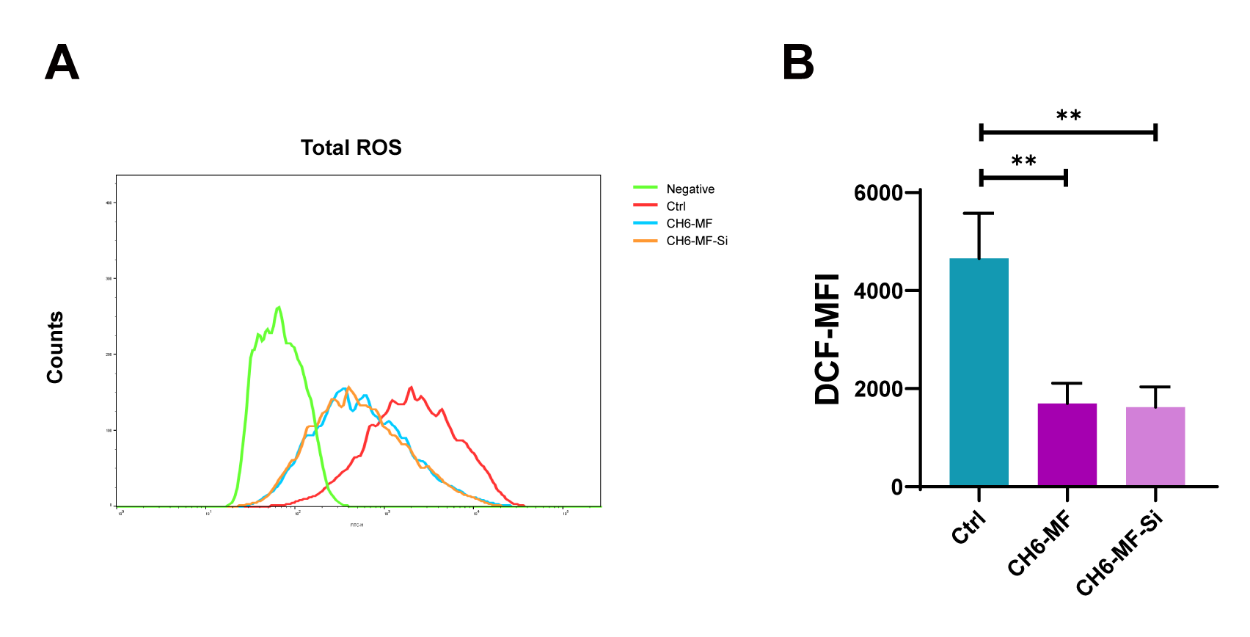


Figure S3. Anti-ROS effects of NPs on hMSCs without TNF-α in vitro. (A) Cellular flow cytometry analysis of total ROS after treatment with PBS, CH6-MF NPs (10 µg/mL) and CH6-MF-Si NPs (10 µg/mL) for 48 h. (B) Quantitative analysis of ROS via detection of the mean fluorescence intensity (MFI) of DCF.


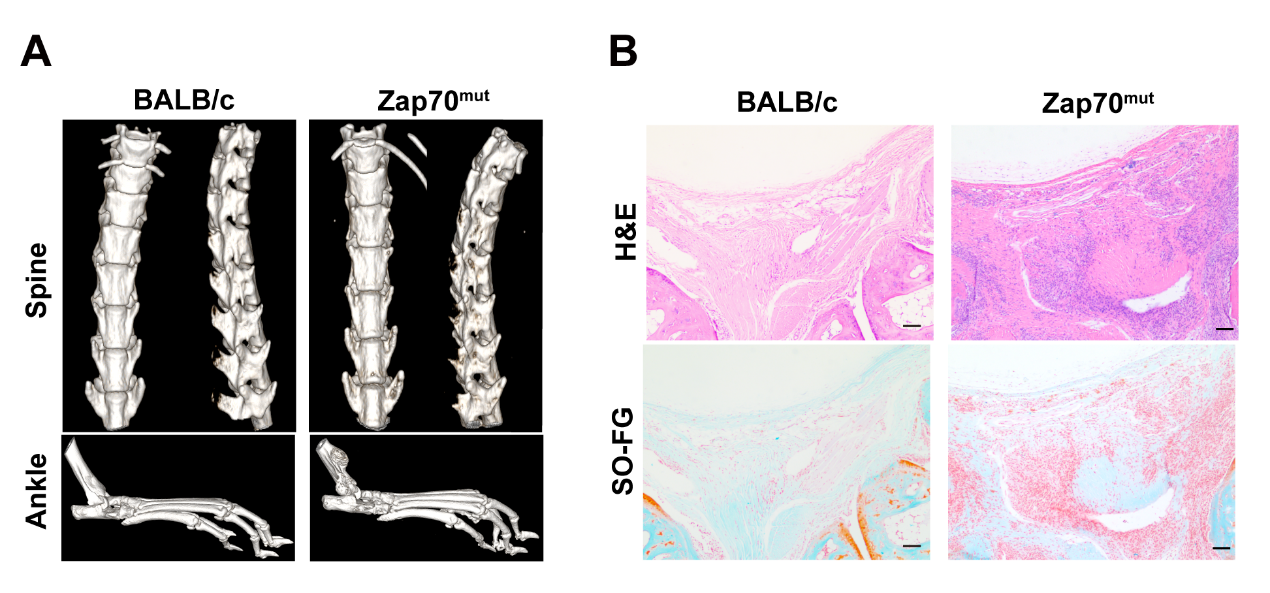


Figure S4. Abnormal osteogenic differentiation and local inflammation of Zap70mut mice. (A) Heterotopic ossification and bone destruction of Zap70mut mice assessed by Three-dimensional micro-CT reconstruction of the spine and ankle. (B) Inflamed ankle joint of Zap70mut mice assessed by haematoxylin-eosin (H&E) and safranin-O (SO-FG) staining.


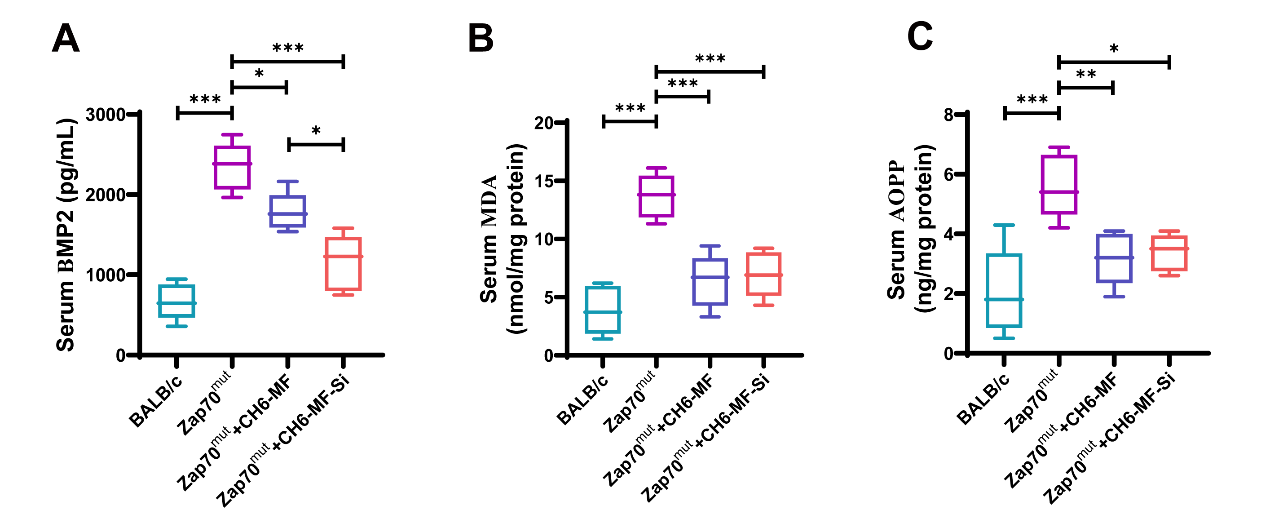


Figure S5. Serum levels of BMP2, MDA and AOPP. (a) The serum levels of BMP2 were measured by ELISA. (b) The serum levels of MDA were measured via an MDA assay kit. (c) The serum levels of AOPP were determined by an AOPP ELISA kit.


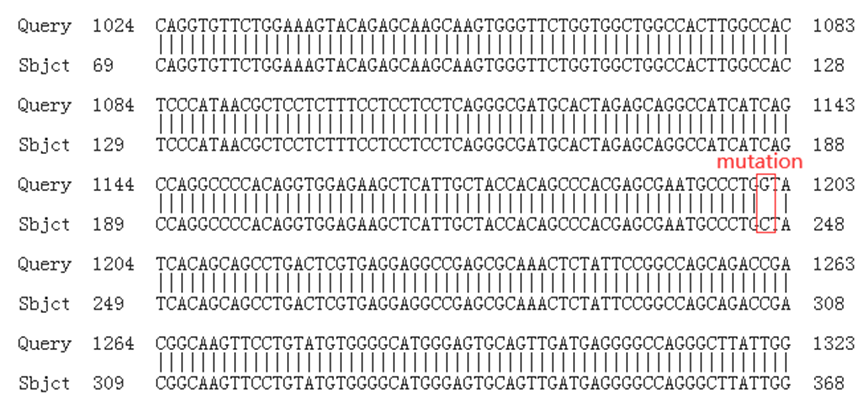


FigureS6. The sequencing information of ZAP70 mutant mice.
